# Oxysterol-binding protein-related protein 4L promotes cell proliferation by sustaining intracellular Ca^2+^ homeostasis in cervical carcinoma cell lines

**DOI:** 10.18632/oncotarget.11671

**Published:** 2016-08-29

**Authors:** Ji-Wei Li, Yan-Lin Xiao, Chao-Feng Lai, Ning Lou, Hong-Ling Ma, Bi-Ying Zhu, Wen-Bin Zhong, Dao-Guang Yan

**Affiliations:** ^1^ Department of Biotechnology, Jinan University, Guangzhou, 510632, China; ^2^ The Key Laboratory of Functional Protein Research of Guangdong Higher Education Institutes, Jinan University, Guangzhou, 510632, China; ^3^ State Key Laboratory of Oncology in Southern China, Collaborative Innovation Center of Cancer Medicine, Guangzhou, 510060, China

**Keywords:** ORP4L, Ca^2+^ homeostasis, NFAT, IP_3_R1, proliferation

## Abstract

Oxsterol binding protein-related protein 4 (ORP4) is essential for cell proliferation, but the underlying mechanism is unclear. ORP4 is expressed as three variants, ORP4L, ORP4M and ORP4S. Here, we reported that silencing of ORP4L with specific small interfering RNA (siRNA) inhibited the proliferation of human cervical cancer cell lines C33A, HeLa and CaSki, the reverse effect being observed in ORP4L overexpressing cells. For molecular insight, we found that ORP4L maintained intracellular Ca^2+^ homeostasis. Through this mechanism, ORP4L activated nuclear factor of activated T cells (NFAT) activity and thus promoted expression of a gene cluster which supported cell proliferation. Of note, ORP4L sustained inositol-1,4,5-trisphosphate receptor 1 (IP_3_R1) expression at both mRNA and protein levels via Ca^2+^-dependent NFAT3 activation, which offered a mechanic explanation for the role of ORP4L intracellular Ca^2+^ homeostasis. Furthermore, ORP4L knockdown markedly inhibited tumor growth in a C33A cell xenograft mouse model. To conclude, our results reveal that ORP4L promotes cell proliferation through maintaining intracellular Ca^2+^ homeostasis.

## INTRODUCTION

Oxysterol binding protein (OSBP)-related proteins (ORPs) comprise a mammalian gene family with 12 members [1] implicated in a spectrum of different cellular processes. The proteins are suggested to act as sterol and phosphoinositide sensors coordinating transport, signaling and metabolism [2–4]. ORP4 (also known as OSBP2) is present as three major variants, ORP4L, ORP4M and ORP4S [5]. ORP4 knockout mice exhibit a teratozoospermia, indicating that ORP4 is essential for the survival of specific cell types [6]. Early studies reported that ORP4L is detectable in peripheral blood leukocytes from patients with chronic myeloid leukemia but not healthy donors [7, 8]. Consistently, recent reports indicate that ORP4L is involved in tumor cell proliferation and survival [5], but the underlying mechanism remains unclear.

Ca^2^^+^ is a versatile second messenger with a wide range of central physiological roles in processes such as cell growth or proliferation [9]. Intracellular Ca^2^^+^ is regulated by an inositol-1,4,5-trisphosphate receptors (IP_3_Rs)-mediated release of Ca^2+^ from the endoplasmic reticular (ER) lumen to the cytosol, which in turn triggers an influx of Ca^2^^+^ across the plasma membrane [10]. IP_3_Rs, including three isoforms (IP_3_R1, IP_3_R2, and IP_3_R3), are membranes of glycoprotein complex acting as a Ca^2+^ channel gated by the secondary messenger inositol trisphosphate (InsP3) [11]. IP_3_R1 is predominant in maintenance of cellular Ca^2+^ signals and oscillations [12]. IP_3_Rs are responsible for the mobilization of Ca^2+^ from intracellular stores in response to signals from receptors on the plasma membrane [13, 14]. One response to the release of Ca^2+^ through IP_3_R is enhanced nuclear factor of activated T cells (NFAT) activity through the Ca^2+^/calmodulin/calcineurin pathway as a consequence of activation of a signal transduction [15–17]. The NFAT family contains five members, including four calcium-responsive isoforms named NFAT1 [18, 19], NFAT2 [20], NFAT3 and NFAT4 [21, 22], and a tonicity-responsive enhancer-binding protein (TonEBP, also known as NFAT5) [23]. They are usually activated by increased intracellular Ca^2+^ levels and subsequently translocated into the nucleus. Once in the nucleus, NFAT1–NFAT4 activate transcription of downstream target genes with multiple regulatory roles in cell fate determination, thus directly linking calcium signaling to gene expression [24–26]. The NFAT isoforms are constitutively activated and overexpressed in several cancer types wherein they transactivate downstream targets that play important roles in cancer cell growth, survival, invasion and angiogenesis [25].

In this study, we offer the first evidences that ORP4L maintains intracellular Ca^2+^ homeostasis and IP_3_R1 expression to facilitate cell proliferation.

## RESULTS

### ORP4L promotes cell proliferation

**Figure 1:**
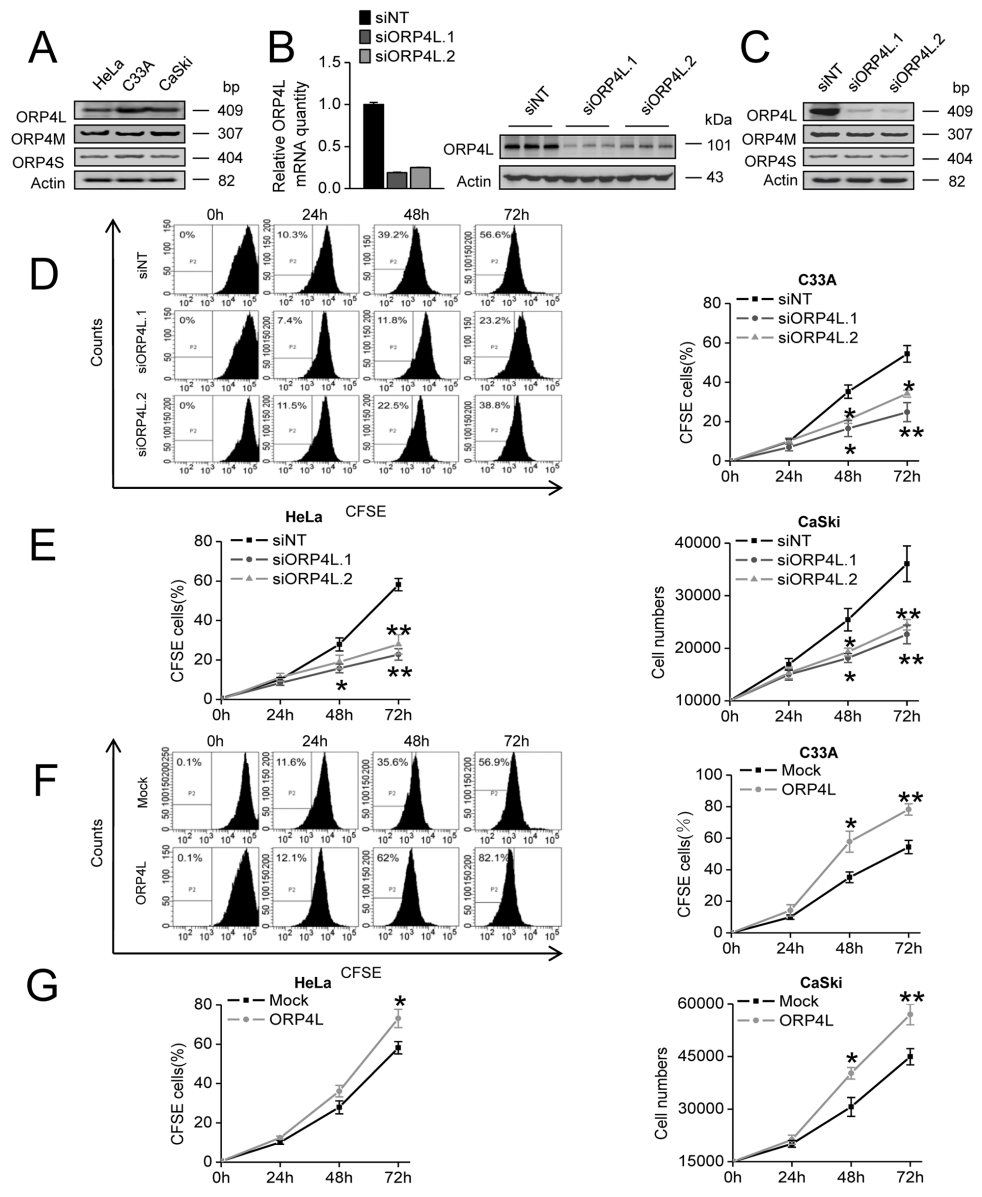
ORP4L is required for C33A, HeLa and CaSki cells proliferation **A.** ORP4/OSBP2 gene transcripts in HeLa/C33A/CaSki cells were detected by RT-PCR. Actin expression was used as an internal control. **B.** HeLa cells were transfected with 60 pmol siORP4L.1, siORP4L.2 or siNT in 12-well plates. Silencing efficiency was accessed at mRNA (qRT-PCR analysis, left panel) and protein level (western blots analysis, right panel). **C.** ORP4L/M/S mRNA was measured by semi-quantitative RT-PCR using cDNAs prepared from HeLa cells transfected with siORP4L.1, siORP4L.2 or siNT. **D.** C33A cells were transfected with siORP4L.1, siORP4L.2 or siNT, flow cytometry analysis of cell proliferation was conducted after 0 hr, 24 hr, 48 hr, and 72 hr with CFSE staining. **E.** HeLa (left) and CaSki (right) cell proliferation upon ORP4L knockdown analyzed by flow cytometry and CCK-8, respectively. **F.** Analysis of C33A cells proliferation after transfection with ORP4L cDNA or empty vector; flow cytometry analysis of cell proliferation was carried out after 0 hr, 24 hr, 48 hr, and 72 hr with CFSE staining. **G.** HeLa (left) and CaSki (right) cell proliferation upon ORP4L knockdown analyzed by flow cytometry and CCK-8, respectively. The data represent mean ± S.D. from three individual experiments (n = 3, *p < 0.05, **p < 0.01).

Previous report shows that the ORP4/OSBP2 gene encodes ORP4L as well as two other variants and is essential for cell proliferation and survival [5]. We found that ORP4L, ORP4M, and ORP4S mRNAs could be detected in human cervical cancer cell lines HeLa, C33A and CaSki (Figure 1A). To specify the role of ORP4L, we analyzed cell proliferation upon ORP4L knockdown or overexpression. We employed two independent siRNAs (siORP4L.1, siORP4L.2) that suppressed only ORP4L expression by targeting exon 1. Compared to a non-targeting control (siNT), both of them reduced ORP4L mRNA and protein expression with silencing efficiencies of approximately 80% in HeLa cells (Figure 1B). Furthermore, we confirmed that siORP4L.1 and siORP4L.2 could only silence ORP4L expression, but did not influence ORP4M and ORP4S mRNA levels as analyzed by RT-PCR (Figure 1C). Then, we treated and analyzed C33A and HeLa cells with CFSE staining upon ORP4L silencing to analyze cell proliferation. ORP4L knockdown significantly reduced C33A (Figure 1D) and HeLa (Figure 1E, left panel) cells proliferation compared to the control. In related experiments, ORP4L knockdown also inhibited CaSki cells proliferation analyzed by CCK-8 assay (Figure 1E, right panel). By contrast, transient overexpression of ORP4L promoted the proliferation of C33A (Figure 1F), HeLa (Figure 1G, left panel) and CaSki (Figure 1G, right panel) cells. These data are consistent with the notion that ORP4L is required for the proliferation of cancer cells.

### ORP4L maintains intracellular Ca^2+^ homeostasis

**Figure 2:**
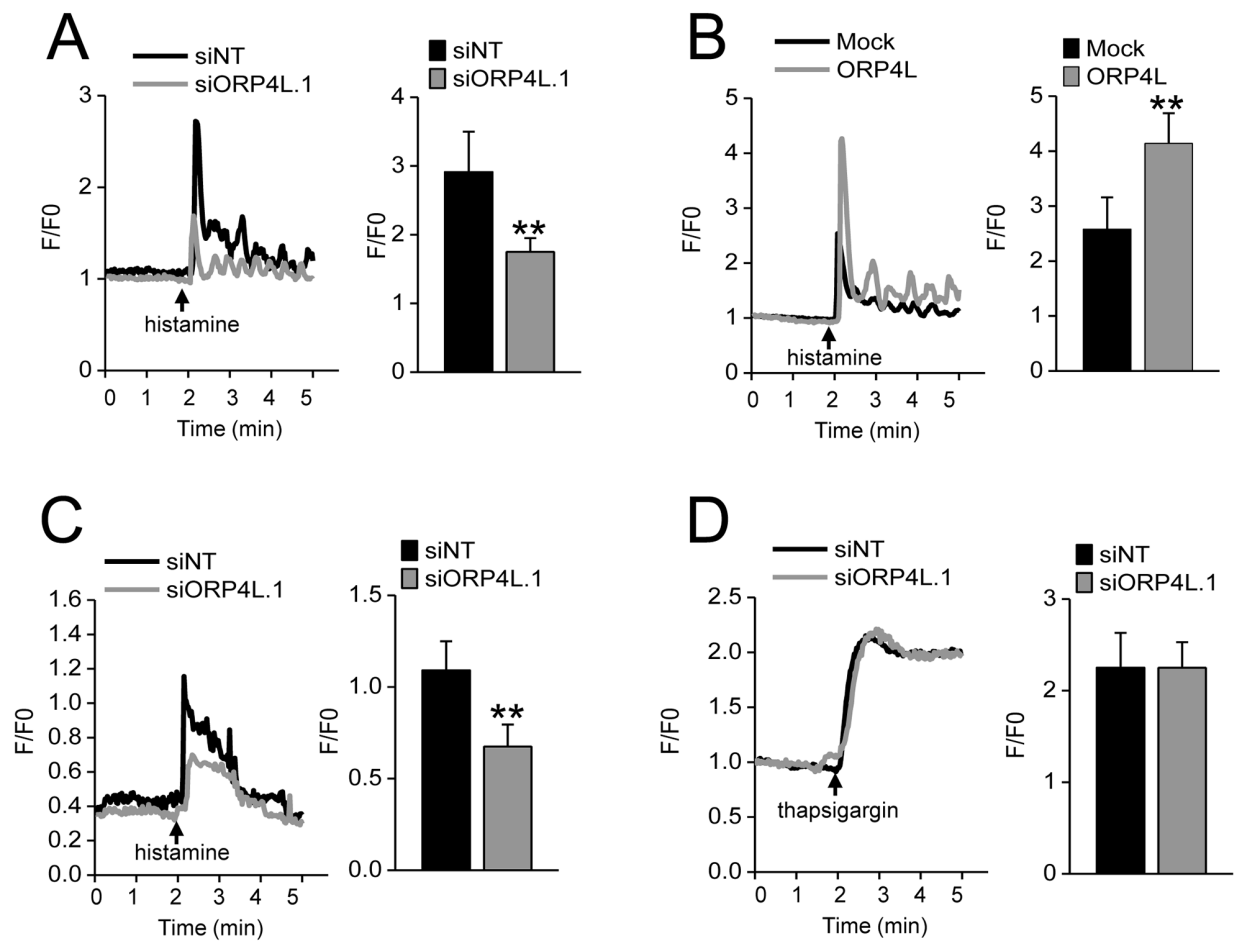
ORP4L maintains intracellular calcium homeostasis **A.** Ca^2+^ release upon 10 μM histamine stimulation in C33A cells subjected to ORP4L knockdown. Changes in intracellular Ca^2+^ were recorded as F/F0 ratio using Fluo4-AM. Representative Ca^2+^ responses and quantification of Ca^2+^ peak amplitudes are shown. **B.** Ca^2+^ release in C33A cells subjected to ORP4L overexpression upon 10 μM histamine stimulation. Changes in intracellular Ca^2+^ are shown. **C.** C33A cells with ORP4L knockdown stimulated with 10 μM histamine in calcium-free medium. Changes in intracellular Ca^2+^ are shown. **D.** C33A cells with ORP4L knockdown stimulated with 1 μM thapsigargin. Changes in intracellular Ca^2+^ are shown. The data represent mean ± S.D. from three individual experiments (n = 3, **p < 0.01).

The role of Ca^2^^+^ in promoting cell proliferation and cell death has been well illustrated and modeled in cancer cells [27]. The function of ORP4L was explored by a Ca^2+^ fluorometry assay of peak calcium elevation after stimulation with histamine [28] in C33A cells subjected to ORP4L knockdown or overexpression, in a buffer containing 1.5 mM CaCl_2_. ORP4L knockdown significantly reduced the peak amplitude as compared to the control (Figure 2A), whereas ORP4L overexpression significantly enhanced this peak amplitude (Figure 2B). To specifically address the role of ORP4L in Ca^2+^ release from ER stores, we analyzed the Ca^2+^ flux in calcium-free medium. The results revealed in the ORP4L knockdown cells also display reduced intracellular Ca^2+^ release, supporting the view that the primary target of ORP4L manipulation was Ca^2+^ release from the ER (Figure 2C). Importantly, ORP4L knockdown did not correlate with thapsigargin induced Ca^2+^ flux that bypasses the IP_3_R1 by shutting down the ER SERCA pump leading to store-operated Ca^2+^ entry (SOCE) (Figure 2D). Together, these data suggest that stable levels of ORP4L protein are required for intracellular Ca^2+^ homeostasis.

### ORP4L increases Ca^2+^-dependent NFAT activity and target genes expression

**Figure 3:**
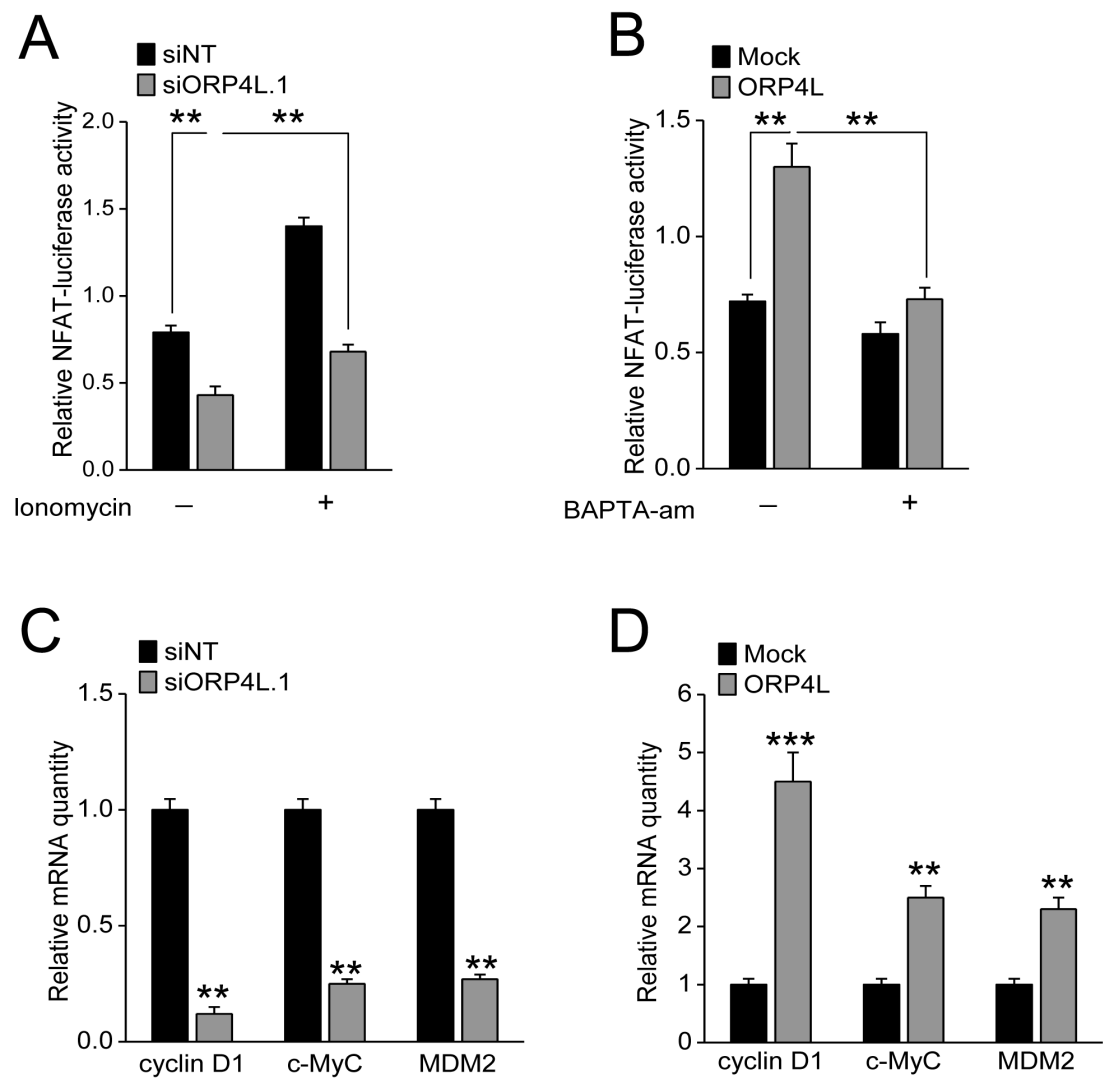
ORP4L increases Ca^2+^-dependent NFAT activity and promotes expression of a gene cluster which supports cell proliferation **A** and **B.** C33A cells were transfected with ORP4L cDNA or ORP4L siRNA, then transfected with pGL4.74[hRluc/TK] and pGL4.30[luc2P/NFAT-RE/Hygro] vectors, treated with or without 2 mg/L ionomycin and 50 μM BAPTA-am for 1 hr, and then measured with Dual-Glo^®^ Luciferase Assay System to determine NFAT activity. **C** and **D.** C33A cells were transfected with ORP4L cDNA or siRNA; relative cyclin D1, MDM2, c-Myc mRNA levels were measured by qRT-PCR. The data represent mean ± S.D. from three individual experiments (n = 3, **p < 0.01, ***p < 0.001).

A generally accepted concept is that NFAT is activated upon Ca^2+^ release and activates expression of a cluster genes promoting cell proliferation [29]. Our observations showed that ORP4L knockdown significantly reduced NFAT activity in C33A cells, which could be partly rescued by the Ca^2+^ ionophore ionomycin (Figure 3A). ORP4L overexpression enhanced NFAT activity in these cells, while this enhanced NFAT activity was significantly decreased by the Ca^2+^ chelator BAPTA-am (Figure 3B). Several studies have revealed how NFATs control cell proliferation by regulating cyclin D1, MDM2, and c-Myc transcription [29]. Our qRT-PCR experiments revealed that cyclin D1, MDM2, c-Myc mRNA expression were significantly decreased upon ORP4L silencing (Figure 3C) and robustly increased in ORP4L overexpressing cells (Figure 3D). These results provide mechanistic evidence that ORP4L maintains Ca^2+^ homeostasis and NFAT activity to promote cell proliferation.

### ORP4L sustains stable expression of IP_3_R1 via activating NFAT3 activity

**Figure 4:**
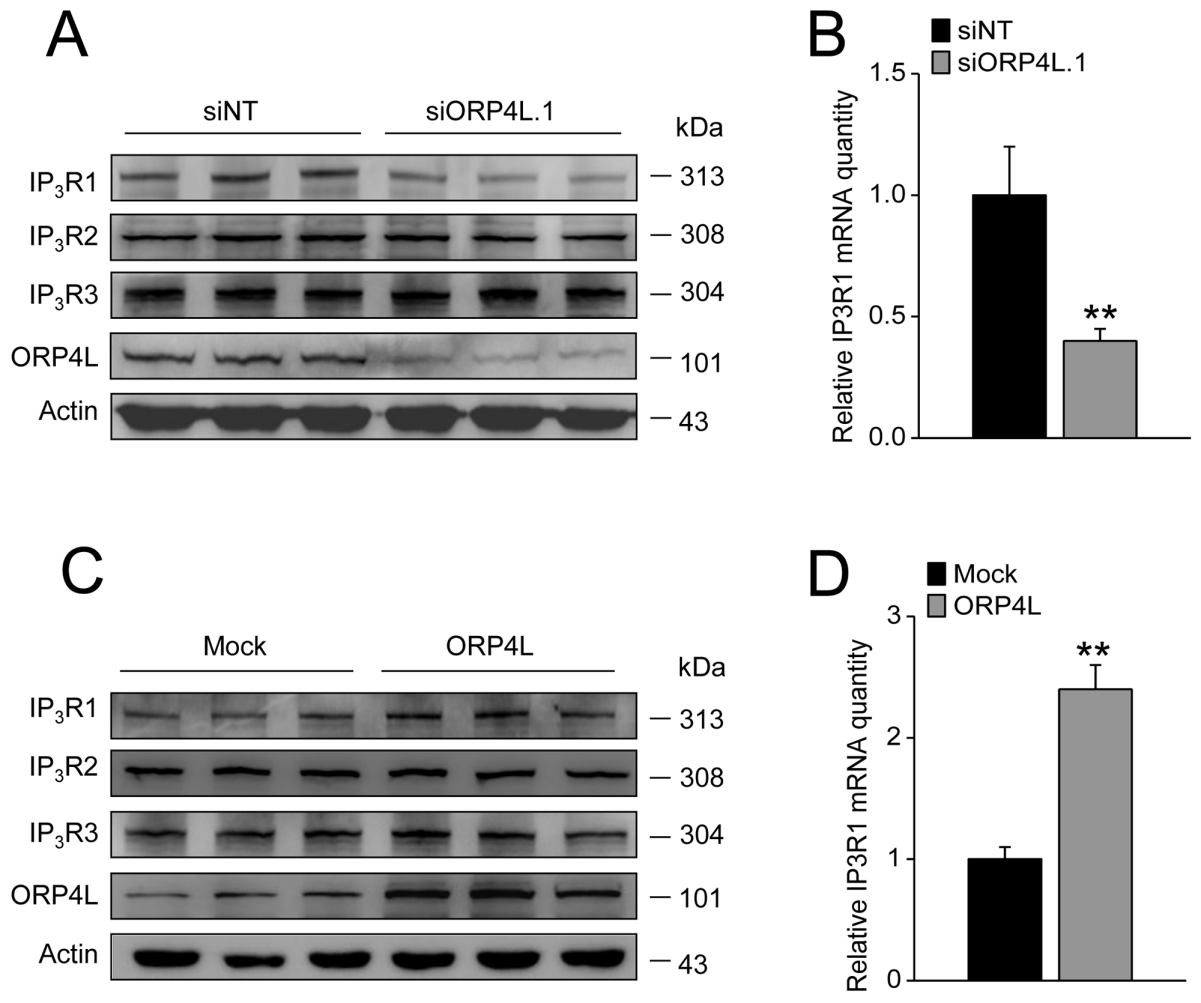
ORP4L maintains stable expression of IP_3_R1 both at protein and mRNA level **A.** and **C.** C33A cells were transfected with 60 pmol siORP4L.1 or 1.6 μg ORP4L cDNA in 12-well plates, western blot analysis of IP_3_R1, IP_3_R2, IP_3_R3 protein levels upon ORP4L knockdown and overexpression, respectively. **B.** and **D.** qRT-PCR analysis of IP_3_R1 mRNA level in C33A cells upon ORP4L knockdown and overexpression, respectively. The data represent mean ± S.D. from three individual experiments (n = 3, **p < 0.01).

IP_3_Rs are the major intracellular Ca^2+^ release channels in cells [30]. IP_3_Rs are also plastic proteins which can be regulated by many factors including the Ca^2+^-regulated NFAT family [31, 32]. We carried out a protein expression analysis of IP_3_Rs in C33A cells with ORP4L knockdown or overexpression. The results demonstrated that ORP4L silencing led to a striking reduction of IP**_3_**R1 protein (Figure 4A) and mRNA expression (Figure 4B). In contrast, ORP4L overexpression was accompanied by increased IP**_3_**R1 protein and mRNA expression (Figure 4C and 4D). However, modification of ORP4L expression did not correlate with IP_3_R2 and IP_3_R3 protein expression (Figure 4A and 4C).

**Figure 5:**
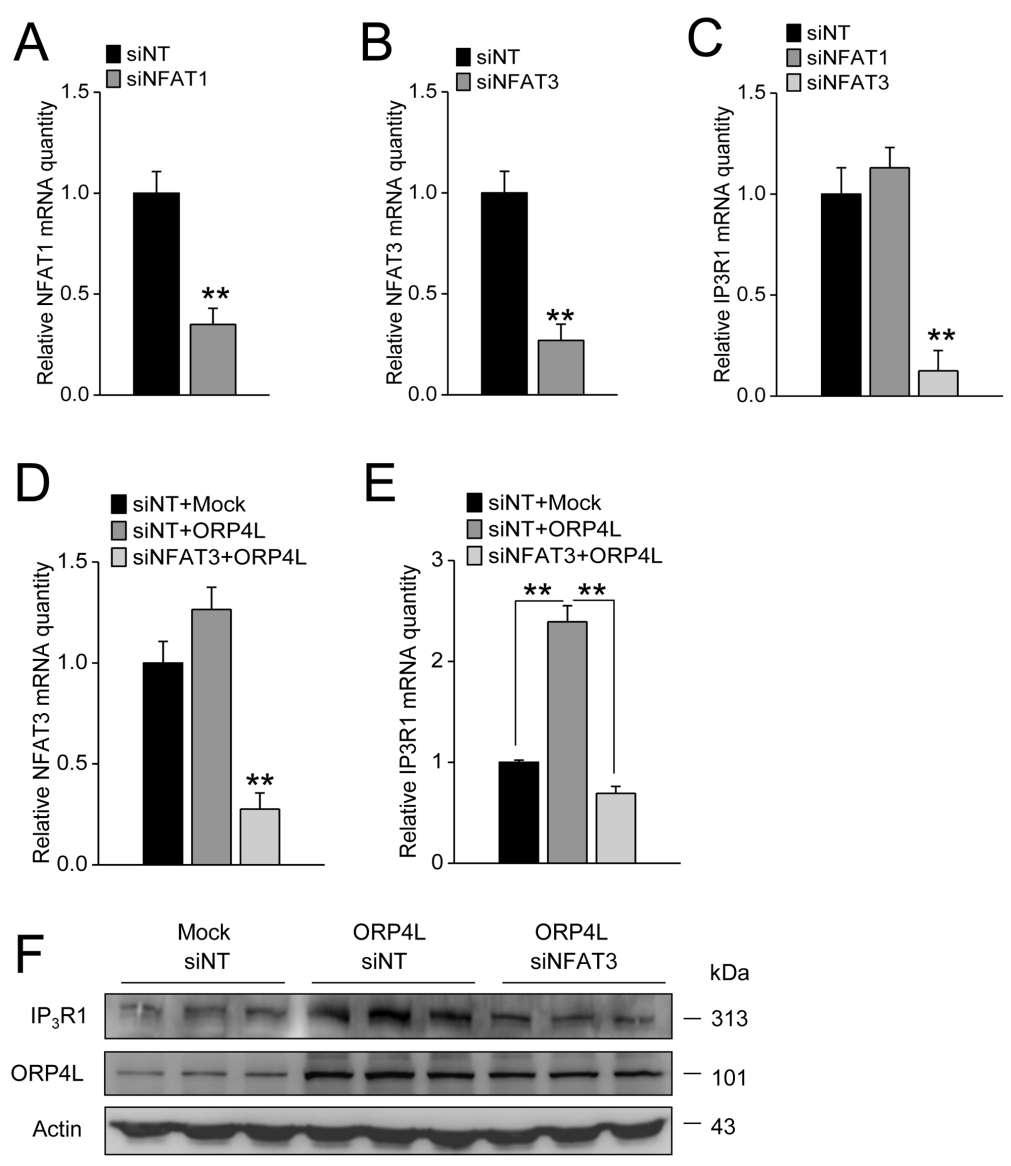
ORP4L maintains IP_3_R1 expression via regulating NFAT3 **A.** and **B.** C33A cells weretransfected with 60 pmol siNFAT1 and siNFAT3 in 12-well plates, qRT-PCR analysis of siNFAT1 and siNFAT3 knockdown efficiency. **C.** IP_3_R1 mRNA levels in C33A cells with NFAT1 or NFAT3 knockdown. **D.** NFAT3 knockdown efficiency in C33A cells transfected with empty vector combined with siNT, ORP4L combined with siNT, and ORP4L combined with siNFAT3. **E.** and **F.** The relative IP_3_R1 mRNA and protein levels in C33A cells treated as in panel D. The data represent mean ± S.D. from three individual experiments (n = 3, **p < 0.01).

Previous reports have already shown that IP_3_R1 expression is predominantly regulated by NFAT3 [31], and NFAT1 could also bind to the IP_3_R1 promoter to induce its transcriptional activation [33]. To investigate the role of NFAT3 and NFAT1 in IP_3_R1 transcriptional regulation in C33A cells, we first employed NFAT1 and NFAT3 siRNAs. qRT-PCR revealed that these siRNAs reduced NFAT1 and NFAT3 mRNA expression effectively (Figure 5A and 5B). NFAT3, but not NFAT1 knockdown significantly reduced IP_3_R1 mRNA expression (Figure 5C), indicating NFAT3 participated in the transcriptional regulation of IP_3_R1 in these cells. To further determined whether ORP4L maintained IP_3_R1 expression via regulating NFAT3 activity, we transfected NFAT3 siRNA and ORP4L cDNA into C33A cells simultaneously. qRT-PCR revealed that the NFAT3 mRNA level was markedly reduced under these conditions (Figure 5D). Expectedly, OPR4L overexpression could not elevate IP_3_R1 mRNA (Figure 5E) and protein (Figure 5F) expression levels in NFAT3 knockdown cells. Taken together, these results suggest that ORP4L maintains IP_3_R1 expression via increasing NFAT3 activation.

### ORP4L knockdown suppresses tumor cell growth *in vivo*

**Figure 6:**
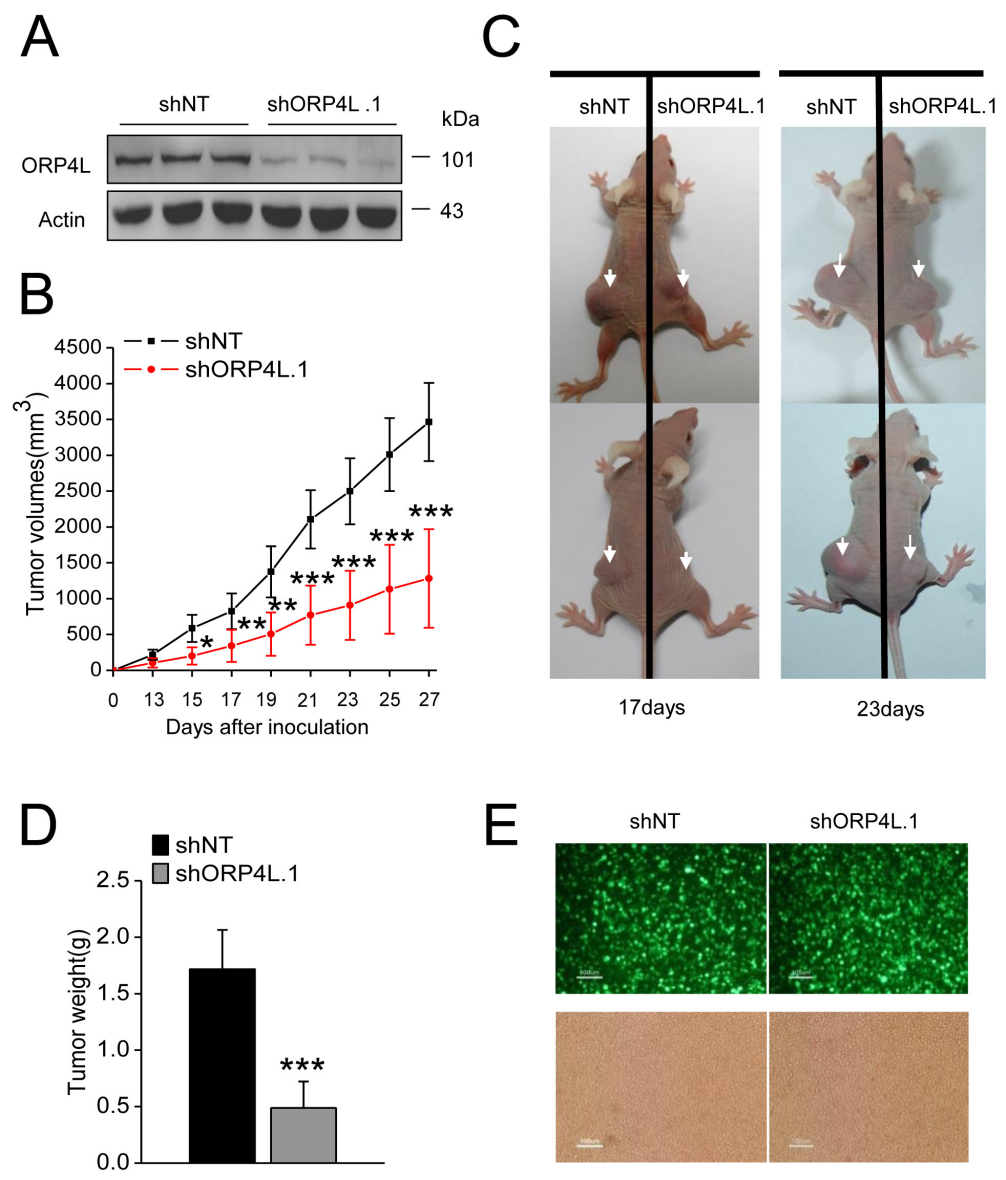
Knockdown of ORP4L in C33A cells inhibits tumor growth **A.** Western blot analysis of knockdown efficiency of ORP4L in C33A cell lines stably transfected with 4 μg shORP4L.1 or shNT in 6-well plates. **B.** Tumor growth over the experiment time course expressed as tumor volume. **C.** Representative examples of animals injected with stable C33A cell lines in the opposite sides, at 17 or 23 days after xenografting are shown. **D.** Tumor weight after excision on day 27. **E.** Visualization of ZsGreen fluorescence in cell suspension from the solid tumor tissue of nude mice. Fluorescence (top) and phase contrast (bottom) images are shown. Scale bars, 100 μm. The data represent mean ± S.D. (n = 7 mice of shNT group, n = 5 mice of shORP4L.1 group, *p < 0.05, **p < 0.01, ***p < 0.001).

In order to study the effects of ORP4L on the *in vivo* tumorigenicity of tumor cells, C33A cells were employed for xenograft experiments. C33A cells were transfected with vectors carring GFP and non-targeting small hairpin RNA (shNT) or ORP4L specific small hairpin RNA (shORP4L.1) and selected with G418 to generate stable cell lines. The ORP4L knockdown efficiency was confirmed by western blotting (Figure 6A). Xenograft experiments carried out for up to 27 days and tumor volume during the entire time course were detected every two days. 7/8 of mice injected with shNT cells and 5/8 of mice injected with shORP4L.1 cells developed subcutaneous solid tumors. We analyzed successfully xenograft animals and results revealed that the tumor growth rate of shORP4L.1 cells was markedly reduced compared to the shNT control (Figure 6B). Examples of the xenografted mice at days 17 and 23 are displayed (Figure 6C). At day 27, mice were sacrificed and tumors were isolated. Tumor weight at day 27 was significantly reduced in the shORP4L.1 side (Figure 6D). GFP fluorescence from the shRNA expression vector was observed in the solid tumor tissues, demonstrating the development of tumor body from the C33A cells administered (Figure 6E). The above results indicate that ORP4L knockdown results in tumor growth arrest *in vivo*.

## DISCUSSION

One of the characteristics of tumor cells is infinite proliferation. ORP4 is selectively expressed in testis, brain and heart but is virtually absent from other human and mouse tissues [6, 34]. Recent reports show that ORP4 knockout results in apoptosis of postmeiotic spermatids in mice, and ORP4 is involved in cell proliferation and survival [5, 6]. Although these studies report important discoveries, the molecular basis of these effects has remained unclear. The reduction in intracellular Ca^2+^ release and subsequently in NFAT activity and the target genes expression in response to ORP4L knockdown reported here provide molecular evidence that ORP4L promotes proliferation through maintaining intracellular Ca^2+^ homeostasis.

*The ORP4/OSBP2* gene encodes ORP4L as well as two truncated variants ORP4M and ORP4S transcribed from alternate transcription start sites. In HEK293 cells, a similar growth arrest effect was observed upon silencing ORP4L and all three ORP4 variants. Reduction of ORP4L expression significantly inhibited HEK293 cell proliferation but only partially suppressed HeLa cell growth [5]. In our experiments, C33A and CaSki cell lines presented more sensitive than HeLa cells to inhibition of proliferation in response to ORP4L knockdown. Although we did not conduct *ORP4M* and *ORP4S* manipulations, the functional observations with siRNAs that specifically target the ORP4L variant, as well as ORP4L overexpression, suggest that this variant plays a dominant role in cell proliferation via targeting Ca^2+^ homeostasis.

Changes in the levels of intracellular Ca^2+^ provide dynamic and highly versatile signals that control cellular processes. Cells generate their Ca^2+^ signals by using both internal and external sources of Ca^2+^ [35]. The internal stores are held within the membrane systems of the ER, which is a substantial reservoir of stored Ca^2+^, and is the principal Ca^2+^ store mobilized for signaling [36]. Transcription downstream of Ca^2+^ flux is in large part funneled through the NFAT transcription factors. NFATs reside within the cytoplasm are in an inactive, phosphorylated form. Following an increase in cytosolic Ca^2+^, activated calcineurin dephosphorylates NFATs, allowing them to enter the nucleus and increase expression of their target genes [37]. In this study, we demonstrate that ORP4L correlates with Ca^2+^ release from ER stores and consequently NFAT activity and target genes expression. These results at least partly account for the effects of ORP4L on cell proliferation noted previously.

IP_3_Rs are a molecule at which many signaling pathways converge and are thus important for generation of versatile Ca^2+^ signaling patterns. Three IP_3_R subtypes have different biophysical properties and are subject to different modulations [12]. When determining the relative expression levels of each IP_3_R subtype, we found that only IP_3_R1 expression was correlated with ORP4L. Previous reports have shown that IP_3_R1 mRNA expression is predominantly activated by NFAT3 [31], NFAT1 could also bind to the IP_3_R1 promoter to induce transcriptional activation of IP_3_R1 [33]. Here we demonstrated that NFAT3 but not NFAT1 could directly increase IP_3_R1 expression in C33A cells. These results suggested that IP_3_R1 was the target gene activated by NFAT3 in C33A cells. The ORP4L-dependent upregulation in IP_3_R1 could result in a greater flux of Ca^2+^ from the ER and elevated levels of total Ca^2+^ released in response to IP_3_-dependent signal transduction. Expression of ORP4L resulted in increased expression of IP_3_R1 leading to an increase in the Ca^2+^ release in response to IP_3_ and in the activity of Ca^2+^-responsive transcription factors.

ORP4L is in early studies reported to be detected in peripheral blood leukocytes from patients with chronic myeloid leukemia but not healthy donors [7, 8]. It is also implicated as a potential marker for solid tumor dissemination and poor prognosis [7]. Also, the Human Protein Altlas (http://www.proteinatlas.org/) suggests that ORP4L is expressed in most cancer cell lines. Moreover, a series of natural products termed ORPphilins exert their antiproliferative effects on cancer cells by targeting ORP4L and its close relative OSBP. These observations, together with the marked reduction of tumor growth rate upon ORP4L knockdown in a C33A cell xenograft model in our study, suggest that ORP4L acts as a pivotal supporter of cell proliferation in cancer cells. This protein may thus be a putative new candidate target for the development of cancer treatment.

## MATERIALS AND METHODS

### Ethics statement

This study has been conducted in accordance with the ethical standards and according to the Declaration of Helsinki and according to national and international guidelines and has been approved the institutional ethics committee of Jinan University.

### Tumorigenicity assays in nude mice

All animals received humane care according to the criteria outlined in the “Guide for the Care and Use of Laboratory Animals” prepared by the National Academy of Sciences and published by the National Institutes of Health (NIH publication 86-23 revised 1985) and according to our institutional ethical guidelines for animal experiments. Four-week-old male BalB/C athymic (nu/nu) nude mice were purchased from the animal center of Guangdong Province (Guangzhou, China) and kept under pathogen-free conditions in the Laboratory Animal Center, Jinan University. The animals were adapted to new conditions for 1 week before the experiments.

An aliquot (1.5 × 10^7^/200μl) of stably transfected shNT and shORP4L.1 C33A cells in PBS were injected subcutaneously into left (shNT) and right (shORP4L.1) rear flank of the same female BALB/c athymic nude mouse. Tumor growth was examined every two days over 27 days (n = 8 per group). The tumor volume was estimated according to the following formula: tumor volume (mm^3^) = ab^2^/2, where a = the larger diameter and b = smaller diameter.

### Antibodies and other reagents

Rabbit anti-ORP4L (catalog: HPA021514; lot number: R09314), histamine, ionomycin, BAPTA-am, thapsigargin and Fluo4-AM were from Sigma-Aldrich. Rabbit anti-IP_3_R1 (catalog: sc-28614; lot number: E1413) was from Santa Cruz Biotechnology. Rabbit anti-IP_3_R2 (catalog: AB3000; lot number: LV1598412) was from Millipore, Mouse anti-IP_3_R3 (catalog: 610312; lot number: 51627) was from BD Transduction Laboratories TM, Mouse anti-β-actin (catalog: 60008-1-Ig; lot number: 10001957) was purchased from Proteintech. The secondary goat anti-rabbit-HRP and goat anti-mouse-HRP antibodies were purchased from Bio-Rad (Hercules CA). CellTrace™ CFSE Cell Proliferation Kit and G418 were purchased from Life Technologies. pGL4.74[hRluc/TK] vector, pGL4.30[luc2P/NFAT-RE/Hygro] vector and Dual-Glo® Luciferase Assay System were purchased from Promega.

### cDNA constructs

Sequence of full-length human *ORP4L* (NM_030758) was obtained by PCR amplification from HeLa cell cDNA by using primers specified in Table 1, and inserted into pcDNA4/HisMaxC vector (Invitrogen). Small hairpin RNAs (shRNAs), shNT and shORP4L.1 were synthesized as oligonucleotides by using primers specified in Table 1, annealed and inserted into vector pSIREN-RetroQ-ZsGreen-Neo respectively. pSIREN-RetroQ-ZsGreen-Neo was derived from pSIREN-RetroQ-ZsGreen (Clontech/Takara Bio, Mountain View, CA) by insertion of Neomycin-ploy A expression cassette downstream of 5′CMV/MSV hybrid promoter. This vector can be selected by G418 resistance and detected via expression of ZsGreen.

**Table 1:** The oligonucleotide primers used

Gene (used for)	Forward primer 5′- 3′	Reverse primer 5′- 3′
ORP4L (RT-PCR, qRT-PCR)	CGTTAAAGCCCCTGCCTCTTCTGC	GTGTTCATCACGCGGACAGCCTTG
ORP4M (RT-PCR)	GAAGCGCCTTGGCATGAACCGTAG	GTGTTCATCACGCGGACAGCCTTG
ORP4S (RT-PCR)	TGGTGTCCTGTGCCATTGTTAAAC	TTGGATGTGATGCGGAAGAGGG
NFAT3 (qRT-PCR)	TTCGGAAGGGTGAGACGGACAT	GCAGGAAGTTGGAGCCAGTCAG
NFAT1 (qRT-PCR)	TGCCTGCCATTCCCATCTGC	CCCTCGGCTGCCTTCTGTCTC
IP_3_R1 (qRT-PCR)	GGCGGAGCAGGGTATTGGAACAG	TTCTTGGGCATTCCGCAACCTAC
CyclinD1(qRT-PCR)	CCGTCCATGCGGAAGATC	ATGGCCAGCGGGAAGAC
c-Myc (qRT-PCR)	TCGGAAGGACTATCCTGCTG	GTGTGTTCGCCTCTTGACATT
MDM2 (qRT-PCR)	GGGAGTGATCAAAAGGACCT	CCAAATGTGAAGATGAAGGTTTC
Actin (RT-PCR, qRT-PCR)	GGCATCCTCACCCTGAAGTA	AGGTGTGGTGCCAGATTTTC
ORP4L (Expression vector)	ATTtctagaATGGGGAAAGCGGCGGT	ATTtctagaGTGGCGCTCAGAAGATGTTG GGGCACATATGCCA
shNT	GCATTGGTCGTCTCTATTA	TAATAGAGACGACCAATGC
shORP4L.1	GCAATGGTTTGCTCTCTTA	TAAGAGAGCAAACCATTGC

### Cell culture

C33A, HeLa and CaSki cell lines were obtained from American Type Culture Collection (ATCC, Rockville, MD, USA). C33A cells and HeLa cells were cultured in DMEM supplemented with 10% fetal bovine serum (FBS), pH 7.4, penicillin (100 U./ml), and streptomycin (100 μg/ml). CaSki cells were cultured in RPMI 1640 supplemented with 10% FBS, pH 7.4, penicillin (100 U./ml), and streptomycin (100 μg/ml). Cells were maintained in 5% CO_2_, 37°C.

### Reverse transcriptase-PCR

Total RNA was isolated with TRIzol reagent according to the manufacturer's instructions; RNA samples were reverse transcribed using random hexamer primers in the presence of RNase inhibitor (Takara Bio, Shiga, Japan). PCR primer sets (Table 1) were designed to amplify specific ORP4 isoforms and actin was amplified as endogenous control. PCR conditions were optimized (30 cycles), the products were separated by 1.5% (w/v) agarose gel electrophoresis and visualized with ethidium bromide.

### RNA interference

One day before transfection, cells were plated on 12-well plates at 30–50% confluence, and then transfected with 60 pmol siORP4L, siNFAT1, siNFAT3 or control non-targeting siRNA (siNT) for 72 hr. (siORP 4L.1: GCAAUGGUUUGCUCUCUUATT; siORP4L.2: UCAGAGUCAAGCUCAGGUGUA; siNFAT1: GCCCAU GGUUGAAAGACAAUU; siNFAT3: CCUGAAGCUUC GGAAUUCAUU; siNT: UAGCGACUAAACACAUC AA) by using Lipofectamine 2000 (Invitrogen).

### Quantitative real-time PCR

Total RNA was isolated from cells with TRIzol reagent according to the manufacturer's instructions, RNA samples were reverse transcribed using random hexamer primers in the presence of RNase inhibitor (Takara Bio, Shiga, Japan). qRT-PCR was performed with SYBR Premex EX Taq (Takara Bio) using the 7300 Sequence Detection System (Life Technologies/Applied Biosystems, Carlsbad, CA). The primers used are shown in Table 1. Relative quantification analysis was performed using the ΔΔCt method with actin as endogenous control; Relative gene expression is presented as the ratio of the target gene to reference control.

### Cell proliferation measurement by flow cytometry (FCM) assay

One day before transfection, cells were plated on 12-well plates. After 12 hr culture, the medium was removed, cells were washed twice with PBS and cultured with fresh medium containing 10 μM CellTrace for 30 min at 37°C in the dark. The cells were washed twice with culture medium and incubated for 1 hr with fresh complete culture medium. Then, cells were subjected to transfection for ORP4L overexpression or knockdown by using Lipofectamine 2000. At different time point, cells were collected and analyzed by Becton-Dickinson (Frankin Lanes, NJ) FACSCalibur instrument.

### Cell proliferation assay by CCK-8

Cells were plated in 96-well plates. After 1-3 days in culture, cell numbers were evaluated by Cell Counting kit-8 following the manufacturer's protocol. Cell number was calculated by standard curve method, and the averages of at least three independent experiments are presented.

### Ca^2+^ measurement

Cells (2 × 10^5^ cells/dish) were incubated with 1 μM Fluo4-AM for 30 min at 37°C in extracellular calcium buffer (ECB, 130 mM NaCl, 5 mM KCl, 1.5 mM CaCl_2_, 1 mM MgCl_2_, 25 mM Hepes, pH 7.5, 1 mg/ml BSA, and 5 mM glucose) in dark, replaced with fresh ECB or ECB lacking CaCl_2_ and containing 0.1 mM EGTA for an additional incubation at 25°C for 30 min to permit dye de-esterification. Then, cells were excited with low-intensity 488-nm laser excitation. Images were acquired at 2-s intervals under time-lapse mode by confocal microscope (Zeiss LSM 510 Meta). Fluorescence was collected for 2 min before histamine (10 μM) or thapsigargin (1 μM) were added into the suspension. Image data were subsequently analyzed using ImageJ (National Institutes of Health) and are presented as a ratio of F/F0 in final results, where F0 represents baseline fluorescence intensity of each cell.

### Western blot analysis

Cells were washed twice with ice-cold PBS, scraped from the dishes, and suspended in lysis buffer (50 mM Tris–Cl, pH 8.0, 150 mM NaCl, 0.5 mM MgCl_2_, 10% glycerol, 1% Triton-X100, 0.1% SDS) with protease inhibitor cocktail (Roche Diagnostics, Basel, Switzerland) on ice for 10 min before clearing of the lysates by centrifugation for 5 min at 12000 × g. The supernatants were collected and analyzed by SDS-PAGE. Images were captured using Tanon-5200 and the densitometry of each band was quantified using Tanon Gis software.

### Dual luciferase reporter assay

Cells seeded in 96-well plates were transfected with pcDNA4HisMaxC-ORP4L vector or siORP4L for 24 hr, then cotransfected with pGL4.74[hRluc/TK] vector and pGL4.30[luc2P/NFAT-RE/Hygro] vector. 24 hr later, 75 μl fresh culture medium was replaced. Then 75 μl luciferase reagent was added and incubated for 10 min in the dark, followed by measurement of the firefly luminescence (560-nm). After adding 75 μl Stop/Glo regent and incubation for 10 min the Renilla luminescence (465-nm) was measured.

### Stable cell line generation

For generation of stable C33A cell lines, cells were plated on 6-well plates at 80-90 % confluence, and then transfected with 4 μg vectors encoding shORP4L.1 or non-targeting shRNA (shNT) by using Lipofectamine 2000, followed by selection with 1000 μg/ml G418. Single clones were obtained by limiting dilution and screened for ORP4L expression by Western blotting.

### Statistical analysis

The data are expressed as mean ± S.D. Differences between groups were assessed by one-way analysis of variance (ANOVA) or Kruskall-Wallis when data were not normally distributed (SigmaStat Software Version 3.5). For groups with small n values, or when the values were not normally distributed, the non-parametric Mann-Whitney U test (SPSS16.0 software package) was used.
